# Longitudinal Sex Steroid Data in Relation to Birth Weight in Preterm Boys

**DOI:** 10.1210/clinem/dgac477

**Published:** 2022-08-16

**Authors:** Kerstin Allvin, Carina Ankarberg-Lindgren, Jovanna Dahlgren

**Affiliations:** Gothenburg Pediatric Growth Research Center, Department of Pediatrics, Institute of Clinical Sciences, Sahlgrenska Academy, University of Gothenburg, S-416 85 Gothenburg, Sweden; Region Västra Götaland, Sahlgrenska University Hospital, Department of Neonatology, S-416 85 Gothenburg, Sweden; Gothenburg Pediatric Growth Research Center, Department of Pediatrics, Institute of Clinical Sciences, Sahlgrenska Academy, University of Gothenburg, S-416 85 Gothenburg, Sweden; Gothenburg Pediatric Growth Research Center, Department of Pediatrics, Institute of Clinical Sciences, Sahlgrenska Academy, University of Gothenburg, S-416 85 Gothenburg, Sweden; Region Västra Götaland, Sahlgrenska University Hospital, Department of Pediatric Endocrinology, S-416 85 Gothenburg, Sweden

**Keywords:** infancy, birth weight, androgens, estrogens, mini-puberty, small for gestational age

## Abstract

**Context:**

There is a lack of knowledge on longitudinal sex steroid patterns during infancy, especially for boys born preterm or with low birth weight (LBW).

**Objective:**

To find out whether LBW boys have a disturbed sex steroid profile during infancy.

**Design and setting:**

Population-based longitudinal study performed at Sahlgrenska University Hospital, Gothenburg, Sweden.

**Participants:**

Ninety-eight singleton boys (47 LBW) born at gestational age 32.0 to 36.9 weeks were included. Because of dropout, 83 of the boys were still in the study at 10 months’ corrected age.

**Main outcome measures:**

Serum androgen and estrogen concentrations were analyzed by gas chromatography-tandem mass spectrometry and IGF-I was determined with radioimmunoassay in umbilical cord and at 0, 2, 5, and 10 months’ corrected age.

**Results:**

Serum levels of androstenedione, estrone, and estradiol declined gradually from birth to 10 months corrected age. In both LBW boys and their counterparts, a surge was seen at 2 months’ corrected age (3 months’ chronological age) for testosterone, median (range) 6.5 (2.0-18.9) nmol/L, and in dihydrotestosterone 1.2 (0.4-4.3) nmol/L. At birth, LBW boys had higher median testosterone (0.7 vs 0.4 nmol/L, *P* = 0.019), and at 0 months’ corrected age, both had higher testosterone (5.7 vs 3.5 nmol/L, *P* = 0.003) and dihydrotestosterone (1.2 vs 0.9 nmol/L, *P* = 0.006) than their counterparts. At 10 months’ corrected age, catch-up in weight SD score from birth correlated with testosterone (*rho* = 0.27, *P* = 0.044) and androstenedione (*rho* = 0.29, *P* = 0.027).

**Conclusions:**

Moderately to late preterm LBW boys showed a disturbed sex hormone profile, with elevated concentrations of androgens in early infancy.

During infancy, rapid endocrine changes take place. After birth, the newborn loses the connection to the endocrinologically active placenta, and hormones such as estrogens and progesterone (1), placental growth hormone (2), and human chorionic gonadotropin (3) decrease in the neonate’s circulation. After full-term parturition, involution of the neonate’s adrenals occurs, with a drop in adrenal hormones such as androstenedione, dehydroepiandrosterone sulfate, and cortisol (4). However, in preterm babies, the activity in the adrenal fetal zone continues to the estimated date of delivery (EDD) and then diminishes (5).

Minipuberty in boys is characterized by a postnatal transient activation of the hypothalamic-pituitary-gonadal axis with increasing levels of LH (6), followed by a rise in testosterone, peaking at 1 to 3 months of age, and thereafter declining until 6 months of age (7, 8). The hormonal surge during minipuberty increases the number of Sertoli cells (9), Leydig cells (10, 11), and germ cells (12, 13). Moreover, the testosterone surge correlates with postnatal penile length and growth rate (14) and with testicular descent and growth (15). Minipuberty is believed to be of importance for future fertility. Most likely, IGF-I is involved in male minipuberty because IGF-I levels at 3 months of age are associated with testicular position and testicular distance to pubic bone (16). Furthermore, IGF-I is involved in preserving the immature Leydig cell pool, as well as local steroidogenesis in human immature testes (17).

To our knowledge, there are no previous studies on longitudinal changes in serum androstenedione, testosterone, dihydrotestosterone (DHT), estrone, or estradiol, determined with tandem mass spectrometry (MS)-based method from birth and during the first year of life.

We have previously shown an altered umbilical sex steroid pattern in preterm neonates born small for gestational age (SGA) (18), as well as in SGA males as young adults (19). Based on these findings, we hypothesized that boys born with low birth weight have a disturbed pattern of androgen secretion that begins during infancy. The aim of the study was to evaluate the association between size at birth and changes in sex steroids, quantified with an MS-based method from birth and during infancy in boys. Furthermore, we aimed to investigate the association between growth, IGF-I, and androgen secretion during infancy in boys.

## Materials and Methods

### Participants

The study was population based and performed at Sahlgrenska University Hospital in Gothenburg, Sweden. Between 2002 and 2004, 98 singleton boys born moderately to late preterm at gestational age 32.0 to 36.9 weeks were included. For all participants, EDD had been calculated by ultrasonography performed at gestational weeks 16 through 18. No boys with serious medical conditions, malformations, or chromosomal anomalies were included in the study. There was a dropout during the study period, and at 10 months after EDD, 83 boys were still in the study (Tables 1 and 2).

**Table 1.** Auxological data at birth, 0, 2, 5, and 10 months’ corrected age, comparing boys with birth weight < 2500 g and birth weight ≥ 2500 g

	Birth weight < 2500 g	Birth weight ≥ 2500 g	*P*
**At birth**	n = 47	n = 51	
Gestational age (wk)	34.1 (32.1-36.7)	36.1 (33.7-36.9)	**0.000**
Weight (g)	2255 (1015-2495)	2765 (2500-3885)	**0.000**
Height (cm)	45.0 (36.0-48.0)^*a*^	48.0 (45.0-54.0)	**0.000**
Head circumference (cm)	31.0 (26.0-35.0)	34.0 (31.0-36.0)^*b*^	**0.000**
Weight (SDS)	-1.19 (-5.46 to 0.69)	0.12 (-1.52 to 2.63)	**0.000**
Height (SDS)	-0.76 (-5.72 to 1.42)^*a*^	0.12 (-2.48 to 4.85)	**0.000**
Head circumference (SDS)	-0.63 (-3.39 to 1.68)	0.32 (-1.45 to 2.25)^*b*^	**0.000**
Cesarean delivery (n)	20 (43%)	9 (18%)	**0.007**
**0 mo corrected age**	n = 47	n = 50	
Age (mo corrected)	0.0 (-1.3 to 0.8)	0.0 (-0.8 to 0.8)	0.770
Weight (g)	3350 (2090-4410)^*c*^	3580 (2620-5020)^*d*^	**0.002**
Height (cm)	49.7 (42.9-53.7)^*e*^	51.3 (47.0-58.2)^*f*^	**0.000**
Head circumference (cm)	35.9 (32.3-38.0)^*c*^	36.4 (34.7-40.0)^*f*^	**0.007**
Weight (SDS)	-1.00 (-4.71 to 1.08)^*c*^	-0.24 (-2.15 to 2.49)^*d*^	**0.000**
Height (SDS)	-1.33 (-5.51 to 1.39)^*e*^	-0.15 (-2.30 to -2.30)^*f*^	**0.000**
Head circumference (SDS)	-0.06 (-2.31 to 1.71)^*c*^	0.42 (-0.92 to 2.81)^*f*^	**0.001**
**2 mo corrected age**	n = 46	n = 46	
Age (mo corrected)	2.0 (1.2-3.4)	2.0 (1.5-3.1)	0.681
Weight (g)	5495 (3975-7490)	6130 (4760-8805)^*c*^	**0.000**
Height (cm)	57.2 (52.7-62.1)	59.5 (55.5-67.2)^*c*^	**0.000**
Head circumference (cm)	39.7 (37.2-42.5)	40.3 (37.5-45.3)^*c*^	**0.008**
Weight (SDS)	-0.07 (-2.50 to 2.96)	0.83 (-0.91 to 4.16)^*c*^	**0.000**
Height (SDS)	-0.63 (-3.31 to 2.18)	0.52 (-1.55 to 3.46)^*c*^	**0.000**
Head circumference (SDS)	-0.03 (-1.40 to 2.04)	0.43 (-1.39 to 3.56)^*c*^	**0.011**
**5 mo corrected age**	n = 45	n = 44	
Age (mo corrected)	4.9 (4.2-6.9)	4.8 (4.0-6.8)	0.200
Weight (g)	7510 (5480-11,620)^*e*^	8000 (5990-10,945)	**0.010**
Height (cm)	65.7 (58.0-74.1)^*e*^	66.8 (62.5-71.6)	0.150
Head circumference (cm)	43.7 (41.0-47.4)^*e*^	43.7 (40.2-46.9)	0.327
Weight (SDS)	-0.22 (-3.37 to 3.10)^*e*^	0.51 (-1.55 to 4.03)	**0.006**
Height (SDS)	-0.14 (-3.57 to 2.56)^*e*^	0.28 (-1.83 to 3.59)	**0.029**
Head circumference (SDS)	0.23 (-1.51 to 2.27)^*e*^	0.46 (-1.93 to 3.68)	0.140
**10 mo corrected age**	n = 42	n = 41	
Age (mo corrected)	10.0 (9.0-12.5)	10.0 (9.2-11.1)	0.695
Weight (g)	9225 (6615-13,365)^*g*^	10,060 (8910-16,470)	**0.005**
Height (cm)	74.4 (68.3-83.0)^*g*^	74.5 (68.9-83.1)	0.218
Head circumference (cm)	46.9 (44.3-50.2)^*g*^	47.3 (43.6-51.4)	0.314
Weight (SDS)	-0.49 (-4.31 to 3.32)^*g*^	0.30 (-0.98 to 5.14)	**0.004**
Height (SDS)	0.01 (-2.06 to 2.84)^*g*^	0.35 (-1.95 to 3.56)	0.242
Head circumference (SDS)	0.34 (-1.64 to 2.47)^*g*^	0.66 (-2.49 to 4.19)	0.319

Values are presented as median with range in parentheses, except for cesarean delivery, which shows n with percentage in parentheses. *P* values were calculated with the Mann-Whitney *U* test for all variables except for cesarean delivery, where Pearson χ ^2^ test was used. Boldface indicates *P* values < 0.05.

^*a*^n = 46.

^*b*^n = 50.

^*c*^n = 45.

^*d*^n = 48.

^*e*^n = 44.

^*f*^n = 47.

^*g*^n = 39.

**Table 2.** Endocrinological data at birth (cord blood), at 0, 2, 5, and 10 months’ corrected age, comparing boys with birth weight < 2500 g and birth weight ≥ 2500 g

	Birth weight < 2500 g	Birth weight ≥ 2500 g	*P*
**At birth**	n = 47	n = 51	
Androstenedione (nmol/L)	2.7 (0.2-6.3)^*a*^	3.0 (1.1-7.8)^*b*^	0.070
Testosterone (nmol/L)	0.7 (0.2-3.7)^*c*^	0.4 (0.2-3.3)^*d*^	**0.019**
Dihydrotestosterone (nmol/L)	0.1 (<0.03-0.5)^*c*^	0.08 (<0.03-0.3)^*d*^	0.064
Estrone (pmol/L)	33823 (1855-151,235)^*c*^	69426 (7178-252,882)^*d*^	**0.009**
Estradiol (pmol/L)	13508 (588-63,733)^*c*^	19232 (4586-64,987)^*d*^	**0.036**
IGF-I (µg/L)	34 (3-92)	59 (11-97)	**0.000**
**0 mo corrected age**	n = 47	n = 50	
Age (mo corrected)	0.0 (-1.3-0.8)	0.0 (-0.8-0.8)	0.770
Androstenedione (nmol/L)	1.2 (0.5-2.9)^*b*^	1.1 (0.4-2.5)^*e*^	0.940
Testosterone (nmol/L)	5.7 (0.8-17.0)^*b*^	3.5 (0.5-14.9)^*e*^	**0.003**
Dihydrotestosterone (nmol/L)	1.2 (0.3-3.0)^*b*^	0.9 (0.3-3.2)^*e*^	**0.006**
Estrone (pmol/L)	25 (<9-75)^*b*^	28 (<9-185)^*e*^	0.392
Estradiol (pmol/L)	6 (<2-19)^*b*^	6 (<2-54)^*e*^	0.134
IGF-I (µg/L)	44 (20-96)^*b*^	55 (16-94)^*c*^	0.072
**2 mo corrected age**	n = 46	n = 46	
Age (mo corrected)	2.0 (1.2-3.4)	2.0 (1.5-3.1)	0.681
Androstenedione (nmol/L)	0.6 (0.2-1.4)^*f*^	0.6 (0.3-1.3)^*g*^	0.838
Testosterone (nmol/L)	6.6 (2.0-18.9)^*f*^	6.4 (3.0-15.1)^*g*^	0.355
Dihydrotestosterone (nmol/L)	1.4 (0.4-4.3)^*f*^	1.1 (0.6-3.6)^*g*^	0.285
Estrone (pmol/L)	12 (<9-32)^*f*^	13 (<9-67)^*h*^	0.483
Estradiol (pmol/L)	5 (<2-12)^*f*^	6 (<2-24)^*g*^	0.062
IGF-I (µg/L)	53 (21-84)^*c*^	52 (10-132)^*e*^	0.939
**5 mo corrected age**	n = 45	n = 44	
Age (mo corrected)	4.9 (4.2-6.9)	4.8 (4.0-6.8)	0.200
Androstenedione (nmol/L)	0.3 (0.1-1.0)^*a*^	0.3 (0.1-1.0)^*i*^	0.942
Testosterone (nmol/L)	0.9 (0.1-5.8)^*a*^	1.7 (0.1-5.8)^*i*^	0.544
Dihydrotestosterone (nmol/L)	0.2 (<0.03-1.0)^*f*^	0.3 (<0.03-0.8)^*i*^	0.691
Estrone (pmol/L)	<9 (<9-26)^*f*^	<9 (<9-26)^*j*^	0.446
Estradiol (pmol/L)	3 (<2-19)^*f*^	2 (<2-22)^*i*^	0.792
IGF-I (µg/L)	48 (15-74)^*c*^	34 (14-147)^*a*^	**0.008**
**10 mo corrected age**	n = 42	n = 41	
Age (mo corrected)	10.0 (9.0-12.5)	10.0 (9.2-11.1)	0.695
Androstenedione (nmol/L)	0.2 (0.1-0.6)^*h*^	0.2 (0.1- 0.4)^*k*^	0,231
Testosterone (nmol/L)	0.2 (0.1-0.3)^*h*^	0.1 (0.1-0.3)^*k*^	0.344
Dihydrotestosterone (nmol/L)	<0.03 (<0.03-0.08)^*l*^	<0.03 (<0.03-0.06)^*k*^	0.974
Estrone (pmol/L)	<9 (<9-13)^*h*^	<9 (<9-13)^*m*^	**0.042**
Estradiol (pmol/L)	<2 (<2-8)^*h*^	2 (<2-8)^*m*^	0.912
IGF-I (µg/L)	49 (30-82)^*n*^	45 (11-87)^*o*^	0.118

Values are presented as median with range in parentheses. *P* values were calculated with the Mann-Whitney *U* test. Boldface indicates *P* values < 0.05.

^*a*^n = 36.

^*b*^n = 42.

^*c*^n = 41.

^*d*^n = 44.

^*e*^n = 39.

^*f*^n = 35.

^*g*^n = 34.

^*h*^n = 33.

^*i*^n = 29.

^*j*^n = 27.

^*k*^n = 26.

^*l*^n = 32.

^*m*^n = 24.

^*n*^n = 38.

^*o*^n = 31.

Forty-seven of the boys had a birth weight < 2500 g, defined as low birth weight (LBW) by the World Health Organization (20). Fifteen of the 98 boys were born SGA, defined as birth weight or birth length below -2 SD scores (SDS) according to the Swedish reference for newborns (21), and 13 of these had LBW. Seventeen boys were born after pregnancies complicated by preeclampsia or hypertension, among whom 15 had LBW. Six of the boys were born after pregnancies complicated by diabetes mellitus (5 type 1, 1 type 2); of these, 1 LBW boy was born after maternal diabetes mellitus type 1.

Four boys were born after assisted reproduction: 2 after in vitro fertilization, 1 after sperm insemination, and 2 after induced ovulation. No boy had hypospadias; 3 boys had unilateral cryptorchidism.

### Auxology

Auxological measurements were taken at regular intervals after birth. Because all boys were born moderately to late preterm, the time points at which measurements were taken were corrected according to EDD. The boys were measured at birth, around EDD, and 2, 5, and 10 months thereafter. Weight was measured using digital infant scales. Length was measured with the infant in a supine position on an electronic infant-length board. Head circumference was measured using measuring tape. SDS were calculated using the Swedish growth reference by Niklasson et al (21).

### Blood Sampling

Umbilical venous blood was collected directly after birth in 85 boys. These are a subgroup of a lager cohort, for whom steroid hormones in umbilical venous blood have already been published (18). Venous blood was drawn once a week if the infant was admitted to a neonatal ward after birth. Blood was also collected around EDD, 2, 5, and 10 months thereafter, in the following text referred to as 0, 2, 5, and 10 months corrected age, respectively.

### Mothers

The median maternal age at delivery was 30.5 (range, 19.5-42.8) years and did not differ between boys born with LBW and their heavier counterparts (data not shown). If present, the following conditions were recorded: hypertension (blood pressure > 140/90); preeclampsia (blood pressure > 140/90 and proteinuria after 20 weeks of gestation); maternal diabetes mellitus type 1 or 2 before pregnancy; and pathological oral glucose tolerance test during pregnancy (the latter defined as plasma glucose ≥ 10.0 mmol/L at 2 hours by the European Association for the Study of Diabetes) (22). The European Association for the Study of Diabetes uses a more stringent definition of pathological oral glucose tolerance test than does the World Health Organization (plasma glucose ≥ 11.1 mmol/L at 2 hours). Fourteen mothers were smoking when admitted to the maternity care center, and 7 were smoking at gestational week 30. There was no difference in smoking habits between mothers of boys born with LBW and others.

### Hormone Assays

Serum concentrations of androstenedione, testosterone, DHT, estrone, and estradiol were simultaneously determined by gas chromatography tandem MS, described in detail elsewhere (23). Calibrators (Ceriliant) were sourced from Sigma-Aldrich AB. The lower limit of detection (LOD) was 0.10 nmol/L for androstenedione, 0.10 nmol/L for testosterone, 0.03 nmol/L for DHT, 9 pmol/L for estrone, and 2 pmol/L for estradiol. Total coefficient of variation (CV) for androstenedione was 17% at 0.5 nmol/L and 13% at 2 nmol/L; for testosterone, CV was 16% at 0.3 nmol/L and < 10% at > 1.5 nmol/L; for DHT, it was 15% at 0.06 nmol/L and 10% at 0.2 nmol/L; for estrone, it was 33% at 11 pmol/L and 14% at 38 pmol/L; and for estradiol, it was 19% at 8 pmol/L and 6% at ≥ 36 pmol/L. However, in 114 of 295 (39%) of the blood samples, the sera volumes were not large enough for the ordinary protocol of 200 µL. Instead, volumes of 100 to 175 µL were used and LOD adjusted accordingly. The reduced sample volumes caused uncertainty in the low ranges close to or below LOD but did not affect results in the higher intervals. Samples with volumes < 100 µL were not accepted for analyses.

Serum IGF-I concentrations were measured using a specific radioimmunoassay (RIA) (Mediagnost GmbH, Tübingen, Germany). The IGF-I samples were diluted 1:50. The measurement range was 3.9 to 250 µg/L. The total CV was 18% at 40 µg/L and 11% at 225 µg/L. The method has been described in detail elsewhere (24).

### Statistical Analyses

Data are presented as median (range). In calculations, hormone concentrations below LOD were assigned the value LOD/2. The Mann-Whitney *U* test was used for comparisons between groups. The Wilcoxon matched-pairs signed-rank test was used for 2 dependent samples. Correlation analyses were performed using Spearman (*rho*) nonparametric rank correlation and *R*^2^. A *P* value < 0.05 was considered significant. The software IBM SPSS Statistics for Windows, version 25.0 (IBM Corp, Armonk, NY, USA) was used for statistical analyses. Figures were drawn using SPSS or Origin version 9.0 (OriginLab Corporation, Northampton, MA, USA).

### Ethical Considerations

The study was approved by the Ethics Committee of the Medical Faculty of the University of Gothenburg (approval number Ö-562-01). Informed consent was obtained from the parents of the participants.

## Results

### Auxological Data

As expected, LBW boys were younger, lighter, shorter, and had smaller head circumference at birth (Table 1). During infancy, LBW boys showed gradual catch-up, and at 10 months’ corrected age, the only difference in auxology seen between groups was in weight (grams and SDS) (Table 1). For all boys studied, catch up in weight SDS from birth correlated with testosterone (*rho* = 0.27, *P* = 0.044, *R*^2^ = 0.04), and with androstenedione (*rho* = 0.29, *P* = 0.027, *R*^2^ = 0.11) at 10 months’ corrected age.

### Androstenedione

**Figure 1.**
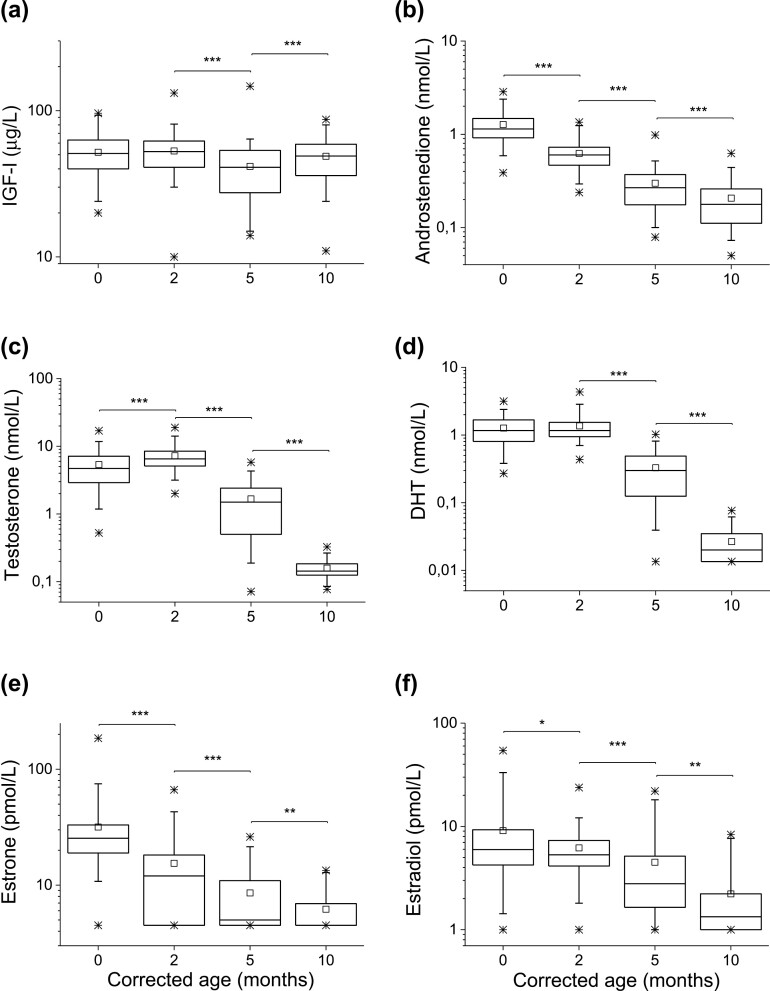
Serum concentrations of IGF-I (A), androstenedione (B), testosterone (C), dihydrotestosterone (D), estrone (E), and estradiol (F) at 0, 2, 5, and 10 months’ corrected age for boys born moderately to late preterm. Box plots represent the 25th, median, and 75th percentile, and whiskers represent the 5th and 95th percentile. Squares represent mean values, and dots represent minimum and maximum values. Each age group includes hormone determinations in between 57 and 97 boys from the original 98 longitudinally followed infants. ***P < 0.001; **P < 0.01; *P < 0.05.

**Figure 2.**
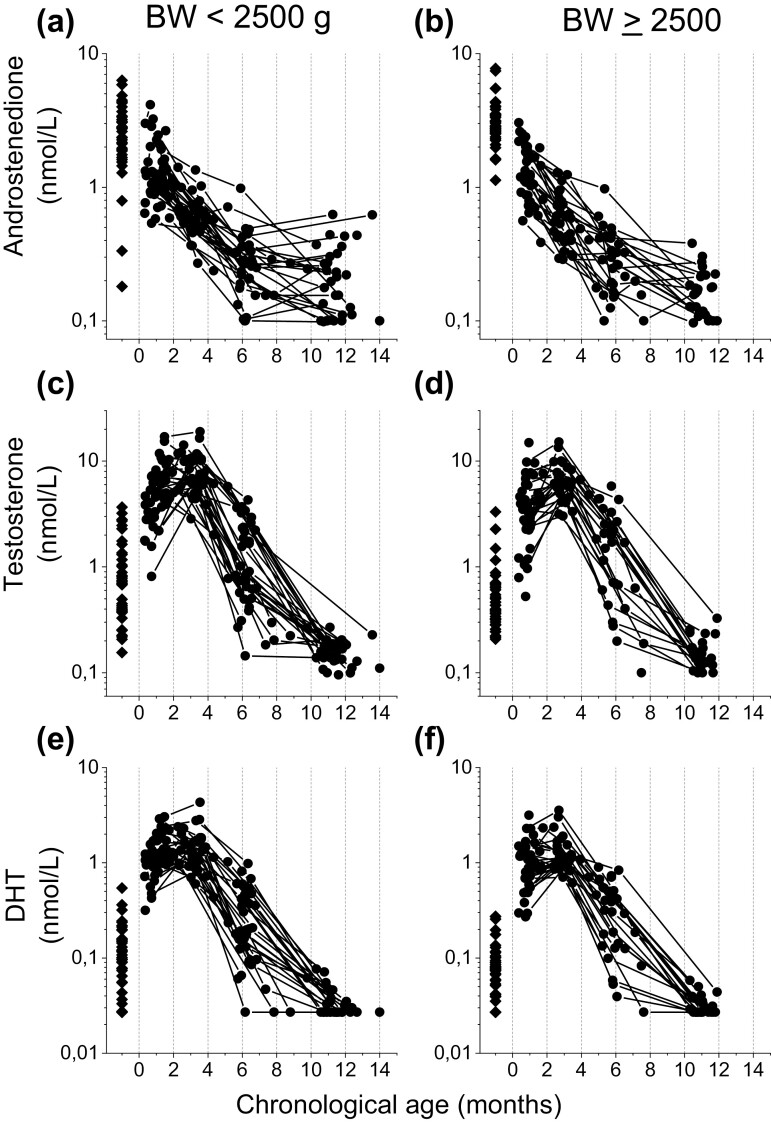
Individual longitudinal serum concentrations for androstenedione (A, B), testosterone (C, D), and dihydrotestosterone (E, F) in moderately to late preterm boys. The left panel shows 47 male neonates born with low weight, defined as birth weight below 2500 g and the right panel shows 50 neonates born with a birth weight of 2500 g and above. The horizontal axis shows chronological age in months. Diamond symbols depict hormone levels in cord blood, whereas circle symbols show hormone concentrations in serum.

Androstenedione concentrations declined continuously from median (range) 1.1 (0.4-2.9) nmol/L at 0 months’ corrected age to 0.6 (0.2-1.4) nmol/L at 2 months, followed by a decline to 0.3 (0.1-1.0) nmol/L at 5 months, and to 0.2 (0.1-0.6) nmol/L at 10 months (*P* = 0.000 for all time points) (Fig. 1B). Thus, androstenedione showed an inverse correlation with corrected age (*rho* = -0.867, *P* = 0.000, *R*^2^ = 0.44). In relation to chronological age, the same pattern was seen with declining levels from birth to about 6 months of age (Fig. 2A and 2B). Androstenedione levels did not differ between LBW boys and their counterparts (Table 2).

### Testosterone

In umbilical cord, LBW boys showed higher median testosterone than their counterparts did (0.7 vs 0.4 nmol/L, *P* = 0.019) (Table 2). Testosterone thereafter increased from 4.7 (0.5-17.0) nmol/L at 0 months’ corrected age to a peak value of 6.5 (2.0-18.9) nmol/L at 2 months, and then declined to 1.5 (0.1-5.8) nmol/L at 5 months, and further to 0.1 (0.1-0.3) nmol/L at 10 months (*P* = 0.000 for all time points) (Fig. 1C). In relation to chronological age, the same pattern was seen but with peak levels at around 3 months, which declined to about 6 months of age (Fig. 2C and 2D). Testosterone at 0 months showed a negative correlation to birth weight (*rho* = -0.40, *P* = 0.000, *R*^2^ = 0.17) and to SDS weight (*rho* = -0.38, *P* = 0.000, *R*^2^ = 0.18). At that age, LBW boys had higher median testosterone than their counterparts did (5.7 vs 3.5 nmol/L, *P* = 0.003) (Table 2).

### Dihydrotestosterone

The concentrations of DHT and testosterone were strongly correlated at 0 months (*rho* = 0.91, *P* = 0.000, *R*^2^ = 0.79), 2 months’ (*rho* = 0.86, *P* = 0.000, *R*^2^ = 0.82), 5 months’ (*rho* = 0.95, *P* = 0.000, *R*^2^ = 0.87), and 10 months’ (*rho* = 0.45, *P* = 0.000, *R*^2^ = 0.20) corrected age. At 2 months’ corrected age (3 months’ chronological age), a peak in DHT was seen at 1.2 (0.4-4.3) nmol/L (Fig. 1D). However, DHT concentrations were statistically unchanged between 0 and 2 months’ corrected age: 1.2 (0.3-3.2) nmol/L vs 1.2 (0.4-4.3) nmol/L (*P* = 0.138). DHT thereafter declined to 0.3 (<0.03-1.0) nmol/L at 5 months (*P* = 0.000) and to < 0.03 (<0.03-0.08) nmol/L at 10 months (*P* = 0.000) (Fig 1D, 2E and 2F). Furthermore, DHT showed the same pattern as testosterone, with a negative correlation to birth weight (*rho* = -0.38, *P* = 0.000, *R*^2^ = 0.11) and to weight SDS (*rho* = -0.40, *P* = 0.000, *R*^2^ = 0.12) at 0 months. At that age, LBW boys had higher median DHT than their counterparts (1.2 vs 0.9 nmol/L, *P* = 0.006) (Table 2).

### Estrogens

**Figure 3.**
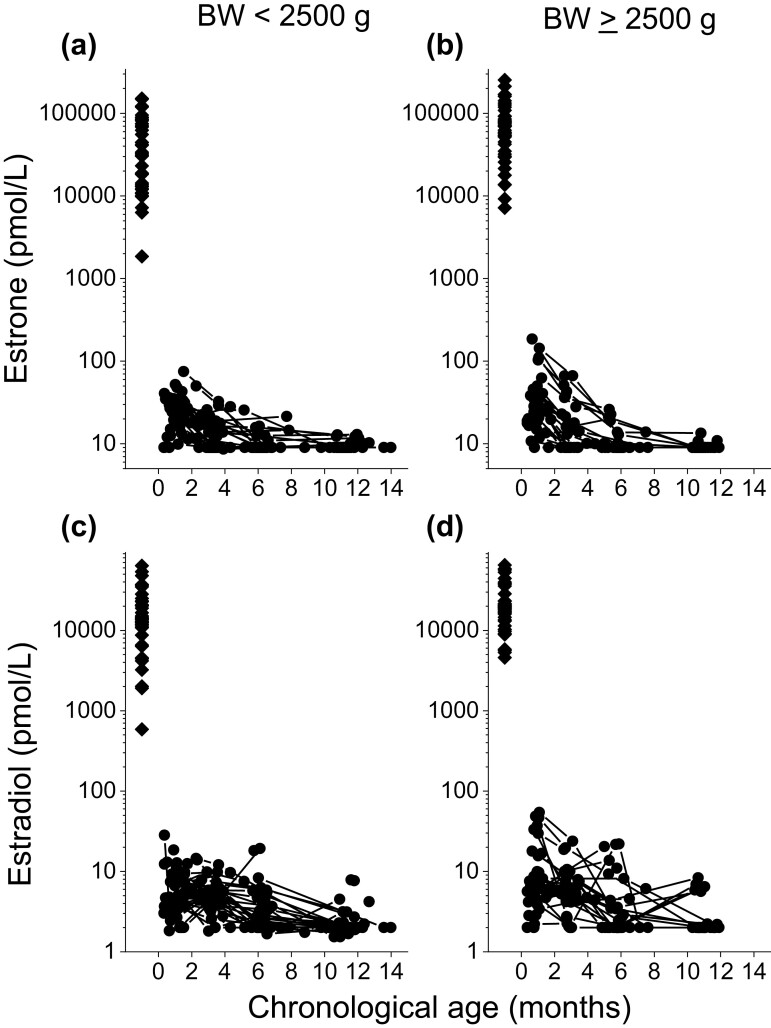
Individual longitudinal serum concentrations for estrone (A, B) and estradiol (C, D) in moderately to late preterm boys. The left panel shows 47 male neonates born with low weight, defined as birth weight below 2500 g and the right panel shows 50 neonates born with a birth weight of 2500 g and above. The horizontal axis shows chronological age in months. Diamond symbols represent hormone levels in cord blood, whereas circle symbols depict hormone concentrations in serum.

Estrone concentrations gradually declined from 25 (<9-185) pmol/L at 0 months’ corrected age to 12 (<9-67) pmol/L at 2 months (*P* = 0.000), to < 9 (<9-26) pmol/L at 5 months (*P* = 0.000), and to < 9 (<9-13) pmol/L (*P* = 0.007) at 10 months (Fig 1E, 3A and 3B).

The same decline was found in estradiol concentrations: 6 (<2-54) pmol/L at 0 months, 5 (<2-24) pmol/L at 2 months (*P* = 0.016), 3 (<2-22) pmol/L at 5 months (*P* = 0.000), and < 2 (<2-8) pmol/L at 10 months (*P* = 0.002) (Fig 1F, 3C and 3D). At birth, LBW boys had lower median concentrations of estrone (33,823 vs 69,426 pmol/L, *P* = 0.009) and estradiol (13,508 vs 19,232 pmol/L, *P* = 0.036) than their counterparts (Table 2). The statistical difference in estrone concentrations at 10 months is most likely irrelevant.

### IGF-I

IGF-I was unchanged between 0 months and 2 months: 51 (20-96) µg/L vs 52 (10-132) µg/L, (*P* = 0.954); it decreased to 41 (14-147) µg/L at 5 months (*P* = 0.000) and increased again to 49 (11-87) µg/L at 10 months (*P* = 0.000) (Fig. 1A).

At 0 months’ corrected age, IGF-I showed a correlation to weight SDS (*rho* = 0.54, *P* = 0.000, *R*^2^ = 0.25), and a negative correlation to testosterone (*rho* = -0.42, *P* = 0.000, *R*^2^ = 0.19) and to DHT (*rho* = -0.45, *P* = 0.000, *R*^2^ = 0.17). Similar correlations were seen at 2 months’ corrected age for testosterone (*rho* = -0.29, *P* = 0.017, *R*^2^ = 0.08), and for DHT (*rho* = -0.31, *P* = 0.011, *R*^2^ = 0.09).

## Discussion

We have studied longitudinal changes in serum androstenedione, testosterone, DHT, estrone, and estradiol determined with MS-based method from birth, during infancy in boys. Our results showed a peak in testosterone and DHT at 2 months’ corrected age, corresponding to 3 months’ chronological age. In the postnatal period, we found increased testosterone and DHT levels in boys born with LBW and an inverse correlation between those androgens and weight. Furthermore, we showed a positive correlation between androgens and catch-up growth in weight SDS at 10 months’ corrected age. Serum concentrations of androstenedione, estrone, and estradiol gradually declined after birth.

The now well-established concept of minipuberty in boys, with a postnatal rise in gonadotropins, followed by a testosterone surge at 1 to 3 months of life, and thereafter a decline, was first described in the 1970s (6, 8, 25). To determine testosterone concentrations during infancy, extraction RIA was used (8, 25), which even today is considered an accurate method to use. Recently, Busch et al. published an article based on longitudinal data, which confirmed the minipuberty peak of testosterone in boys, of the same magnitude as our study, determined by liquid chromatography tandem MS (26).

Since the 1970s, several researchers have sought to establish reference intervals for sex steroids during infancy. However, in most studies, sex steroid concentrations were determined by direct immunoassays, known to give falsely elevated concentrations, compared with MS- based methods, in umbilical cord blood, neonates, and infants (27-29). Hence, studies that used immunoassays present 3 to 10 times higher androstenedione concentrations than we did in the present study (4, 30). Because of overestimation and imprecision, several previous studies did not report peak values at 1 to 3 months of age, despite serial measurements on the same patients (30, 31). However, there are a few studies on androgens in infants performed by MS-based methods, based on cross-sectional data, for very and extremely preterm infants (32), full-term infants aged 2 to 5 months (33), and from birth until adulthood (34, 35). All of these reports presented levels of androstenedione and testosterone in the same range as in the present study, but because of the study design, were unable to capture the dynamic changes during infancy.

We found declining androstenedione concentrations from birth until 10 months’ corrected age, in line with a previous study in which adrenals of deceased boys were examined, concluding that androstenedione is mainly synthesized by the adrenals and decreases from birth during the first 2 years of life (36).

In infant boys, testosterone is mainly synthesized in the testes the first 4 months of life, and at 6 months the adrenals take over (36). As shown in previous studies (8, 26), we found a testosterone peak at 2 months’ corrected age or 3 months’ chronological age. Moreover, boys born with LBW had elevated serum testosterone levels in cord blood as well as at 0 months corrected age, supported by a previous animal study showing increased intratesticular testosterone concentrations 20 days’ postpartum in intrauterine growth-restricted rats (37). However, in a previous cohort of boys born SGA, Pepe et al. found that testosterone at 3 months of age was higher in boys born SGA, and in addition was higher in preterm boys (38).

Interestingly enough, studies in the 1980s performed with extraction-RIA showed that both preterm boys and term boys born SGA have a different postnatal testosterone surge than term boys of normal size. Whereas preterm boys presented a higher peak in testosterone, peaking at a higher postnatal age and with a more prolonged decline, term boys born SGA presented with a testosterone peak at the same postnatal age, but the testosterone concentrations remained elevated for a more prolonged period (39, 40). Altogether, LBW and prematurity seem to enhance and prolong the minipuberty period. It therefore seems logical that the boys born with LBW in the present study, who were all preterm, presented with increased testosterone levels around EDD. Moreover, our results are in line with our previous study showing a negative correlation between cord androgen concentrations and birth weight SDS and between cord androgen concentrations and birth length SDS (18).

We can only speculate on the clinical implication of elevated testosterone levels in boys born with LBW. It has previously been shown that growth velocity is highest in boys at 1 month of age, when the testosterone peak during minipuberty is seen (41), indicating the importance of testosterone for growth. Therefore, we hypothesize that not only IGF-I (42), but also testosterone, may be beneficial for optimizing catch-up growth in boys born after intrauterine growth restriction; this is supported by our finding of a positive correlation between catch-up growth in weight and androgen levels at 10 months’ corrected age. A previous study found a positive correlation between urinary testosterone levels and growth velocity during the first 5 months of life (41). Others found that testosterone levels at 4 and 8 weeks of age correlate with body mass index later in life (43), emphasizing the importance of androgen levels at a very young age for growth later in life in boys. In the present study, we found an inverse correlation between IGF-I and androgen levels at 0 months’ corrected age. However, being born with LBW means having both low IGF-I (44) and, as shown in our study, high androgen levels, suggesting that LBW could be a confounder for the inverse correlation between IGF-I and androgens.

To our knowledge, longitudinal changes in DHT during infancy in boys, quantified with MS-based method have not been presented before. Serum DHT followed the same pattern as testosterone, peaking at 2 months’ corrected age and thereafter declining. The DHT concentrations at 0 to 2 months of age were higher in boys with LBW, about 10 times higher than in cord blood (18), and about the same as in young adult males (19).

We present data for the first time on longitudinal serum concentrations of estrone and estradiol, determined with a sensitive and accurate assay, during the first 10 months of life in boys. The levels of estrogens (mainly believed to originate from the placenta) were initially extremely high in cord blood and gradually declined during infancy. Although boys born with LBW displayed lower estrogen levels in cord blood, their heavier counterparts had similar levels of estrogens at all time points studied during infancy. In the present study, the LOD for the estradiol assay was 2 pmol/L, which was necessary to distinguish the decline in estradiol seen during the first months of life, because the majority of samples had estradiol concentrations below 10 pmol/L. Previous studies using direct immunoassay for determination of estradiol in neonates and infants (33, 45, 46) all presented higher values compared with our study. Their estradiol concentrations were most likely falsely elevated because it is well known that direct estradiol immunoassays overestimate low concentrations compared with methods based on tandem MS (47).

The strength of our study is the longitudinal design with regular serial blood sampling up to 10 months’ corrected age in a period where rapid developmental changes occur. However, the crucial strength is the use of an accurate and sensitive laboratory method, gas chromatography tandem MS, for determination of sex steroid concentrations. Furthermore, we present data on the participants’ prenatal environment and their auxological data up to 10 months’ corrected age.

Not all blood sample volumes reached the prerequisite of 200 µL, which forced us to adjust the LOD accordingly. The consequence of the reduced sample volumes was that a larger number of samples resulted in hormone concentrations below LOD, and accordingly with lower precision at low levels. Looking retrospectively at the study design, more frequent sampling would have been preferable, providing us with more precise data about the dynamic changes in sex steroids during infancy in boys.

In conclusion, we show that the well-established rise in testosterone concentration during male infancy is accompanied by a rise in DHT. In contrast, androstenedione, estrone, and estradiol gradually decline after birth. Furthermore, we show elevated testosterone and DHT levels at around estimated time of birth in preterm boys born with LBW, as well as an inverse correlation between levels of IGF-I and testosterone and between levels of IGF-I and DHT concentrations. Finally, a higher degree of catch-up growth in weight from birth to 10 months of age correlates with higher androgen levels at 10 months. Whether our results are also applicable to full-term boys merits further studies.

## Data Availability

Restrictions apply to the availability of some or all data generated or analyzed during this study to preserve patient confidentiality or because they were used under license. The corresponding author will on request detail the restrictions and any conditions under which access to some data may be provided.

## Abbreviations

CV: coefficient of variation
DHT: dihydrotestosterone
EDD: estimated date of delivery
LBW: low birth weight
LOD: limit of detection
MS: mass spectrometry
RIA: radioimmunoassay
SDS: SD score
SGA: small for gestational age
